# Identification of Atrial Fibrillation by Quantitative Analyses of Fingertip Photoplethysmogram

**DOI:** 10.1038/srep45644

**Published:** 2017-04-03

**Authors:** Sung-Chun Tang, Pei-Wen Huang, Chi-Sheng Hung, Shih-Ming Shan, Yen-Hung Lin, Jiann-Shing Shieh, Dar-Ming Lai, An-Yeu Wu, Jiann-Shing Jeng

**Affiliations:** 1 Stroke center and Department of Neurology, National Taiwan University Hospital, Taipei, Taiwan; 2 NTU-NTUH-MediaTek Innovative Medical Electronics Research Center, Taipei, Taiwan; 3 Department of Electrical Engineering, National Taiwan University, Taipei, Taiwan; 4 Department of Internal Medicine, National Taiwan University Hospital, Taipei, Taiwan; 5 Department of Mechanical Engineering, Yuan Ze University, Tao-Yuan, Taiwan; 6 Division of Neurosurgery, Department of Surgery, National Taiwan University Hospital, Taipei, Taiwan

## Abstract

Atrial fibrillation (AF) detection is crucial for stroke prevention. We investigated the potential of quantitative analyses of photoplethysmogram (PPG) waveforms to identify AF. Continuous electrocardiogram (EKG) and fingertip PPG were recorded simultaneously in acute stroke patients (n = 666) admitted to an intensive care unit. Each EKG was visually labeled as AF (n = 150, 22.5%) or non-AF. Linear and nonlinear features from the pulse interval (PIN) and peak amplitude (AMP) of PPG waveforms were extracted from the first 1, 2, and 10 min of data. Logistic regression analysis revealed six independent PPG features feasibly identifying AF rhythm, including three PIN-related (mean, mean of standard deviation, and sample entropy), and three AMP-related features (mean of the root mean square of the successive differences, sample entropy, and turning point ratio) (all p < 0.01). The performance of the PPG analytic program comprising all 6 features that were extracted from the 2-min data was better than that from the 1-min data (area under the receiver operating characteristic curve was 0.972 (95% confidence interval 0.951–0.989) vs. 0.949 (0.929–0.970), p < 0.001 and was comparable to that from the 10-min data [0.973 (0.953–0.993)] for AF identification. In summary, our study established the optimal PPG analytic program in reliably identifying AF rhythm.

Atrial fibrillation (AF) is an important risk factor for systemic and cerebral embolism^1^. Currently, the detection of AF rhythm mainly relies on the clinical symptoms and a short-period electrocardiogram (EKG) exam^2^. Although patients with paroxysmal AF have a stroke risk similar to that of patients with persistent AF, the former are usually asymptomatic, and their condition is often undetected by routine EKG^3^,^4^. Thus, longer monitoring or more frequent EKG recordings have been recommended to increase the diagnostic rate forAF^5^,^6^,^7^. But there are some limitations of EKG-based strategies, such as a short monitoring period (24-h Holter EKG), requiring patients to trigger the recorder (the patient-triggered event recorder), and high costs or invasive procedures (the mobile cardiovascular telemetry, the use of external event or loop recorders, or the use of insertable cardiac monitors)^2^,^8^.

Photoplethysmogram (PPG) is an optics-based technology that can detect changes in blood flows during the heart’s activities and has been empirically applied to measure the saturation of oxygen and heart rate as pulse oximetry^9^. Compared to EKG procedures, obtaining PPG signals is much easier and more convenient and can be measured from fingertips, wrists, or earlobes by simple and portable devices at any time and occasion^10^,^11^,^12^. Therefore, if PPG signals associated with AF rhythms can be reliably differentiated from those from non-AF rhythms, monitoring PPG signals may have potential for use in screening and identifying patients with AF, especially for those with paroxysmal AF.

In the present study, we prospectively collected the continuous waveforms of EKG and PPG signals simultaneously in patients admitted to the stroke intensive care unit (ICU). We aimed to investigate whether quantitatively analyzing PPG waveforms can clearly identify patients with AF; we especially focused on selecting the PPG features and appropriate data length of PPG for feature extraction to optimize the PPG analytic program for AF identification.

## Results

### Study Subject Demographics

After excluding patients with pacemaker rhythm or poor signal quality (n = 35) and non-persistent EKG rhythm (n = 2), a total of 666 stroke patients were recruited into analysis. Among them, 150 patients (22.5%) were labeled as AF, and 516 patients (77.5%) were labeled as non-AF. The clinical information for the study subjects is presented in Table 1. Compared to AF patients, non-AF patients were younger, had a higher percentage of hypertension, and had a lower National Institutes of Health Stroke Scale (NIHSS) score (all p < 0.05).

### Establishing Independent PPG Features and Optimal Data-collection time for AF Identification

In univariable analysis, the values of most PPG features showed significant differences between AF and non-AF subjects, including those data extracted from 1-, 2-, or 10-min duration of data (Table 2). Furthermore, logistic regression analysis showed 6 independent PPG features that identified subjects with AF, including 3 that were PIN related [mean, mean of standard deviation, and sample entropy (SampEn)], and 3 that were peak AMP related [mean of root mean square of the successive differences, SampEn, and turning point ratio (TPR)] (all p < 0.01) (Table 3).

**Figure 1:**
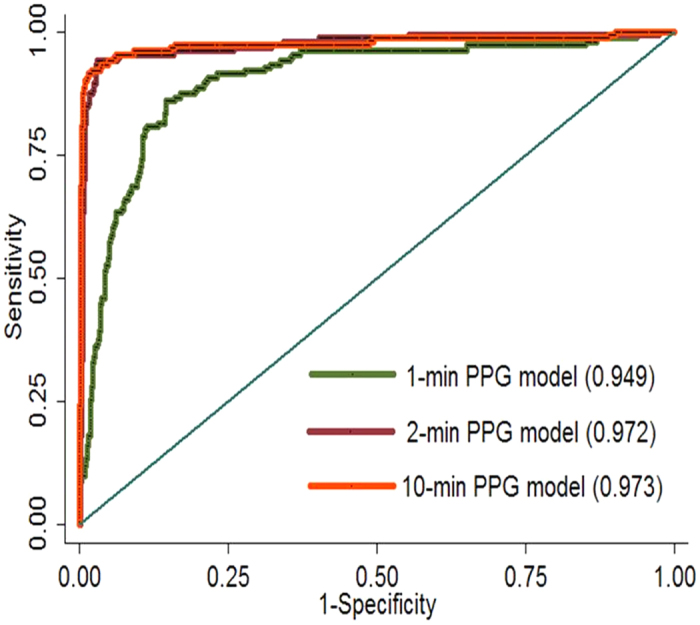
The performances of PPG models comprising the six independent PPF features extracted from the one, two and 10-minute data for AF identification were compared. The area under the ROC curves for the 2-minute PPG model was significantly higher than that for the one-minute [AUC = 0.972 (95% confidence interval 0.951–0.989) and 0.949 (95% confidence interval 0.929–0.970), p < 0.001], and comparable to the 10-minute model [AUC = 0.973 (95% confidence interval 953–0.993)].

The performances of PPG analytic programs that included these 6 features for AF identification, extracted from the first 1-, 2-, and 10-min data, are shown in Fig. 1. The area under the ROC curves for the 2-min PPG analytic program was significantly higher than that for the 1-min [AUC = 0.972 (95% confidence interval 0.951–0.989) and 0.949 (95% confidence interval 0.929–0.970), respectively, p < 0.001] and was comparable to that for the 10-min analytic program [AUC = 0.9730 (95% confidence interval 953–0.993)]. Moreover, the sensitivity, specificity, positive predictive value, negative predictive value, and accuracy of AF identification for the 2-min PPG analytic program were 94.0, 96.8, 89.8, 98.2, and 96.2, respectively.

## Discussion

Currently, short-term EKG examination and 24-h EKG Holter monitoring are the 2 most common methods for the diagnosis of AF, though the detection rate is not ideal in patients with paroxysmal AF^8^,^13^. Recently, one randomized trial showed that a 30-day event-triggered recorder can have a significantly higher rate of AF detection than conventional 24-h Holter EKG monitoring (16.1% vs. 3.2%) in patients with cryptogenic stroke^6^. Another randomized trial compared the use of an insertable cardiac monitoring device to determine the rate and time of the first detection of AF within 6 months and conventional follow-up in patients with cryptogenic stroke. The results indicated that the proposed strategy was much superior to the conventional method (8.9% vs. 1.4%)^7^. Therefore, a longer duration of EKG monitoring or frequent intermittent EKG recording has been recommended in subjects with suspected paroxysmal AF^14^.

However, the use of insertable EKG devices or long-term wearing of an EKG event recorder do have some limitations in clinical practice. Thus, the need for an additional modality other than EKG for AF detection based on emerging methods and technologies is truly important^15^,^16^. Recently, several clinical studies demonstrated the utility of automated oscillometric blood pressure (BP) monitors equipped with an AF detection program for screening AF during routine BP measurement^17^,^18^,^19^. According to the preprogrammed instructions, the automated BP monitor would measure the last ten PIN during the cuff deflation-phase of a BP measurement and thus would determine the regularity of the time intervals.

Nevertheless, the convenience of oximetry is considerable, and the PIN data can be obtained from PPG signals more easily and reliably than can data from an oscillometric BP monitor, especially in terms of the frequency of sampling and long-term monitoring. Besides, not only PIN information but also PPG signals can provide information about AMP, which can be affected concurrently by cardiac arrhythmia. From the literature, we found that applying PPG signals for AF detection has been reported only by a research group that used a smart phone built-in camera lens to obtain the PPG in a small number of study subjects^5^,^12^,^20^. The analytical algorithm used contained only features that originated from PIN but not AMP in the PPG signals. In our study, the logistic regression analysis clearly demonstrated the independent role of AMP features in constructing the optimal PPG analytic program for AF identification.

Recently, several nonlinear analytical methods had been applied to quantify the complex regulatory dynamics of human biological signals such as heart rate variability^21^, electroencephalography^22^, and intracranial pressure^23^. Because the characteristics of those signals are physiologically nonlinear, the advantages of using nonlinear methods vs. conventional linear methods to describe the complex patterns have been shown in several earlierstudies^21^,^22^,^23^,^24^. Our study showed that the optimal PPG analytic program for AF detection included both linear and nonlinear features, which indicates a synergistic rather than a competitive relationship between the linear and nonlinear PPG features.

In addition to identifying the features for measurement, we also investigated the optimal duration of time for PPG data collection and tested 3 different durations for PPG data collection, namely,1, 2, and 10 min. Theoretically, the shorter time required for PPG data collection would imply easier and broader clinical application. By contrast, longer times for data collection may ensure the stability and quality of the calculated values, especially for nonlinear features^25^. Our results suggest that 2 minutes is the optimal amount of time for sampling to achieve a balance between efficiency and convenience. Most importantly, because PPG signals can be continuously obtained long periods, for example, during a whole night of sleep, the PPG analytic program we developed can analyze the data every 2 minutes to facilitate the detection of paroxysmal AF.

Nowadays, PPG signals can be obtained easily via some newly developed wearable devices such as smart watches and smart phones. Therefore, our results suggest the potential for collecting PPG signals for use with our proposed analytical model to reliably detect patients with AF. On the one hand, patients with a normal sinus rhythm as shown by routine EKG but with positive AF alarms from our PPG analytic program could indicate the high possibility of paroxysmal AF, and these patients should be encouraged to receive long-term EKG monitoring or frequent intermittent EKG recordings. On the other hand, patients who have negative AF findings during repeated analysis of long-term PPG signals may indicate a lower chance of paroxysmal AF.

Our study results indicate that even the optimal PPG analytic program may still misclassify a certain amount of EKG data in clarification of AF from non-AF rhythm. There may have at least 2 possible causes: First, all PIN features originate from R–R intervals, and thus sinus arrhythmia or AF with less irregular R–R intervals may be misclassified. Second, we excluded only a small percentage (<6%) of study subjects because of predefined criteria regarding poor signal quality. Inevitably, the collected signals still contain background noises and motion artifacts that may affect the accuracy of the analytical program. Therefore, any strategy that improves data collection and analysis would theoretically further improve the accuracy of our proposed PPG analytic program for AF identification and detection.

There are several potential limitations to this study. First, we collected EKG and PPG signals only from patients admitted to our stroke ICU, and thus whether our results can be extrapolated to other populations is uncertain, especially because PPG signals are more vulnerable to motion artifacts compared to EKG signals. In our study, since all patients kept lying in bed during the data collection, the moving artifacts for both EKG and fingertip PPG were not visually significant. Nevertheless, because PPG signals can be easily obtained and our proposed PPG analytic program requires only a few minutes of data, frequent intermittent recording or long-term recording while the patient is resting or sleeping should be able to overcome this issue. It is also reasonable to perform additional prospective studies to verify our study results. Further studies with very long period monitoring data may also be considered to verify the reliability of our proposed PPG program. Second, there were some differences in clinical parameters between the subjects with and without AF, and the effects of those parameters on our proposed PPG analytic program in AF identification cannot be known. However, the purpose of our study was to develop a purely PPG-based AF detection program without consideration of clinical parameters; therefore, we did not include clinical parameters in the logistic regression model. Third, we used the traditional algorithm of “derivative and threshold” to find the peaks of PPG signals. The precision of peak identification may be largely affected by the quality of PPG signals. However, since the purpose of our proposed PPG program is to differentiate AF from non-AF rhythm, even though the peaks of PPG signals may not be sampled precisely, it wouldn’t easily affect the final result. We will also make effort on the signal processing and algorithm to minimize this potentially analytic inaccuracy in the future. Nevertheless, because of their convenience and practicability, our proposed PPG program and analytical strategy do have potential as a first-line screening tool to detect patients with paroxysmal AF.

## Conclusions

In summary, we performed the first real-world clinical study to investigate the usefulness of PPG signals extracted from routine pulse oximetry in identifying AF rhythms. Our results established the optimal PPG analytic program and its feasibility in reliably identifying patients with AF.

## Methods

### Study Subjects

This study was conducted at the stroke ICU of the National Taiwan University Hospital, with the approval of the Institutional Ethics Committee. Patients who were admitted to the stroke ICU between February 2012 and November 2015 were prospectively recruited. Written informed consent was obtained from the patient or the next of kin for patients with impaired consciousness. All methods were performed in accordance with the relevant guidelines and regulations.

The entry criteria for our stroke ICU included ischemic stroke patients receiving thrombolytic therapies or endovascular treatments, acute stroke patients receiving aggressive blood pressure controls, patients with moderate to severe stroke severity defined as NIHSS score >8, and patients with stroke in evolution or medical conditions that required intensive care^26^. Demographic data were prospectively collected from each recruited patient.

### EKG and PPG Data Acquisition

We set up a standard procedure to collect EKG and fingertip PPG (pulse oximetry) analog data directly from the bedside monitor (Intellivue MP70, Philips, Netherlands) as described in our previous studies^21^,^24^. The EKG and PPG signals were simultaneously collected for at least 10 min with sampling frequencies of 512 Hz and 128 Hz, respectively. Two of the authors (SCT and CSH) reviewed and labeled the 10-min EKG data as AF, non-AF, pacemaker rhythm, or poor signal quality. Heart rates lower than 40 pulses/min or higher than 150 pulses/min or subjects with non-persistent EKG rhythm (for example, AF that changed to non-AF or vice versa) during the 10-min data were excluded. AF rhythm was defined as the absence of P waves with disorganized electrical activity in their place and irregular R–R intervals for >30 s^7^,^27^.

### Signal Preprocessing and Feature Extraction

**Figure 2:**
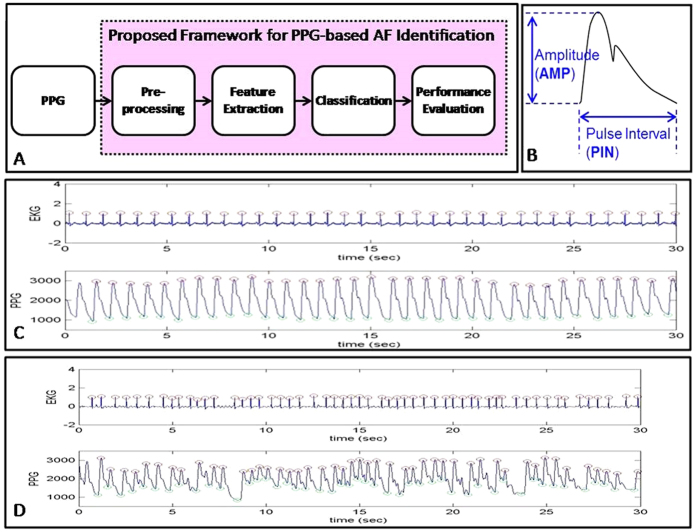
The proposed framework for photoplethysmogram (PPG) based atrial fibrillation (AF) identification (**A**). The parameters of pulse interval (PIN) and amplitude (AMP) can be obtained by analyzing PPG waveform (**B**). The potential of applying PPG parameters to identify AF can be shown in figure **C** and **D** that there are obvious differences of PPG waveforms between AF and non-AF rhythms.

The study framework was shown in Fig. 2A. Briefly, the purpose of the preprocessing was to obtain the parameters of PIN and AMP from the PPG waveforms. The traditional “derivative and threshold” algorithm was used to find the peaks of PPG signals^28^. The original signal is differentiated and the threshold is set as a fixed ratio of the standard deviation of the signal. The high peak and low peak is the largest and smallest value occurring within a data window of fixed length around the position when the signal exceeded the threshold. After the peaks were found, the amplitude (AMP), and pulse interval (PIN) series were extracted from each pulse wave of PPG signals as shown in Fig. 2B. The potential of the PPG signals for AF detection is demonstrated in Fig. 2C and D, which show that the morphologies of the PPG waveform display obvious difference in terms of PIN and AMP between AF and non-AF rhythms.

Various PPG features were extracted from the first 1, 2, and 10 min of measured parameters of PIN and AMP. The features can be divided into 2 types: The first type, extracted with traditional linear analytical methods, reflects the variability of data, including mean, standard deviation, and root mean square successive difference between adjacent data points and reflects time-domain variability, whereas power in the low-frequency range (LF), power in the high-frequency range (HF), and the ratio of LF and HF reflect frequency-domain variability^21^. The second type, extracted with nonlinear analytical methods, reflects the complexity of data, including the SampEn, Shannon entropy, and TPR^21^,^29^. The mathematical equations for the aforementioned are described below.

#### Linear features

We used some of the most popular linear features in heart rate variety researches. Linear Features include mean, standard deviation of mean, root mean square successive difference of mean, power in LF, power in HF as in the reference^21^.

#### SamEn

SamEn is a non-linear method which has been widely used to evaluate the physiologic control mechanisms. The *SampEn* for each series 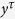 is calculated:


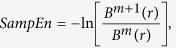


where *m* is the pattern length and *r* is the similarity criterion. Sample entropy is a measure of complexity. *B*^*m*^(*r*) is the number of two sets of simultaneous data points of length m have distance <*r*. So sample entropy is the conditional probability that a dataset, having repeated itself within a tolerance r for m points, will also repeat itself for m + 1 points. Thus, the more complex the signal is, the higher the entropy will be.

#### Shannon entropy

Shannon entropy is a common entropy definition in information theory. Shannon’s measure of information is the probability of symbols to represent the amount of uncertainty or randomness in a data source. We classify parameter series into a fixed number of groups, which is closer to Shannon entropy’s original definition. First, we removed outliers, which had larger difference to mean value than 3 standard deviations. Second, we sorted rest data into equally spaced *N* bins, where *N* is the number of bins. Last, we calculate the probability *p*_*i*_ of each bin and apply Shannon entropy:


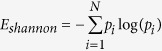


The optimal number of groups is data dependent, so we applied *N* = 4, 8, 16, 32 and 64 for experiment. Shannon entropy quantifies the randomness of overall distribution of each parameter histogram.

#### TPR

TPR is based on the nonparametric “Runs Test” to measure the randomness in a time-series. Each beat in a RRI series is compared to its two nearest neighbors and is defined a turning point if it is greater or less than 2 neighbors. The turning point ratio is the ratio of turning point to total data length *L*. Turning point ratio is higher when series is more random.


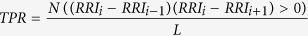


And turning point ratio is applied on PPG parameters in the same way.

### Statistical Analysis

Statistical analysis was performed using R 3.2.4 software (R Foundation for Statistical Computing, Vienna, Austria). The distributional properties of continuous variables were expressed by mean ± standard deviation and median and interquartile range, and categorical variables were presented by frequency and percentage. In univariable analysis, the differences between AF and non-AF data were analyzed by a 2-sample *t* test and a chi-square test. Next, multivariable analysis was conducted using a logistic regression model to estimate the independent PPG features in AF detection. The receiver operating characteristic (ROC) curves were applied to evaluate model performances. The cutoff value for AF detection from the optimal PPG analytic program was determined by the Yuoden index. The sensitivity, specificity, positive predictive value (PPV), and negative predictive value (NPV) of the established PPG analytic program for AF detection were calculated. Sensitivity was defined as the probability of a positive result from PPG analysis if AF was truly positive. Specificity was the probability of a negative result from PPG analysis if AF was truly negative. The PPV was defined as the probability that AF was positive if that the results from PPG model suggestive of AF, while the NPV was the probability that AF was negative if results from PPG model suggestive of non-AF. In statistical testing, a 2-sided p value ≤ 0.05 was considered statistically significant.

## Additional Information

**How to cite this article:** Tang, S.-C. *et al*. Identification of Atrial Fibrillation by Quantitative Analyses of Fingertip Photoplethysmogram. *Sci. Rep.* **7**, 45644; doi: 10.1038/srep45644 (2017).

**Publisher's note:** Springer Nature remains neutral with regard to jurisdictional claims in published maps and institutional affiliations.

## Figures and Tables

**Table 1:** Clinical Characteristics of Study Population.

	All subjects (n = 666)	non-AF (n = 516)	AF (n = 150)	P (non-AF vs AF)
Age, years	66.3 ± 15.1	63.9 ± 14.8	74.5 ± 12.8	<***0.001***
Male	376 (56.6)	297 (57.6)	79 (52.7)	0.304
Diabetes mellitus	239 (35.9)	180 (34.9)	59 (39.3)	0.334
Hypertension	533 (80.0)	413 (80.0)	120 (80.0)	1.000
Hyperlipidemia	291 (43.7)	233 (45.2)	58 (38.7)	0.162
History of stroke	152 (22.8)	110 (21.3)	42 (28.0)	0.097
Smoking habit	376 (56.5)	297 (57.6)	79 (52.7)	0.104
NIH stroke scale	14 (7–20)	12 (6, 18)	18 (14, 25)	

Values are mean ± standard deviation or number (percentage). NIH stroke scale is represented as median ± interquartile range.

**Table 2:** Univariate analysis of PPG features between AF and non-AF subjects.

	non-AF (n = 516)	AF (n = 150)	p value
*Pulse interval*			
Mean	0.797 ± 0.150	0.775 ± 0.134	0.093
Standard deviation of mean	0.265. ± 0.289	0.375 ± 0.247	<0.001
RMSSD of mean	0.357 ± 0.397	0.488 ± 0.324	<0.001
Low frequency	0.012 ± 0.027	0.018 ± 0.051	0.075
High frequency	0.016 ± 0.051	0.025 ± .093	0.111
Sample entropy	0.874 ± 0.518	1.835 ± 0.581	<0.001
Turning point ratio	0.487 ± 0.106	0.596 ± 0.054	<0.001
*Systolic amplitude*			
Mean	1.763E3 ± 254.505	1.376E3 ± 386.567	<0.001
Standard deviation of mean	0.157 ± 0.106	0.316 ± 0.124	<0.001
RMSSD of mean	0.180 ± 0.137	0.435 ± 0.169	<0.001
Low frequency	3.049E4 ± 1.619E4	2.532E4 ± 1.947E4	0.001
High frequency	1.707E4 ± 1.621E4	3.007E4 ± 2.908E4	<0.001
Sample entropy	1.716 ± 0.620	2.320 ± 0.427	<0.001
Turning point ratio	0.564 ± 0.086	0.672 ± 0.061	<0.001

Values are mean ± standard deviation.

AF: atrial fibrillation; RMSSD: root mean square of *successive* differences.

Represented by the 2-minute analytic program.

**Table 3:** Logistic regression of independent PPG features in AF identification.

	Estimate	Std. error	Odds ratio	95% CI	p value
Mean_PIN	−3.631	1.340	0.0265	0.002–0.404	<0.001
Mean SD_PIN	−14.294	2.378	10.787	2.330–49.945	0.008
Sample Entropy_PIN	3.625	0.511	37.519	13.774–102.197	<0.001
Mean RMSSD_AMP	5.471	1.243	237.654	20.786–2717.225	<0.001
Sample Entropy_AMP	1.774	0.524	5.896	2.111–16.469	<0.001
TPR_AMP	10.077	2.523	23792.175	169.268–3.3* 10^6^	<0.001

Values are mean ± standard deviation.

PIN: pulse interval. AMP: systolic amplitude. SD: standard deviation. RMSSD: root mean square of *successive* differences. TPR: turning point ratio.

Represented by the 2-minute analytic program.
